# Cardiac Sarcomere Signaling in Health and Disease

**DOI:** 10.3390/ijms232416223

**Published:** 2022-12-19

**Authors:** Ashley A. Martin, Brian R. Thompson, Dongwoo Hahn, Addeli Bez Batti Angulski, Nora Hosny, Houda Cohen, Joseph M. Metzger

**Affiliations:** Department of Integrative Biology and Physiology, University of Minnesota Medical School, Minneapolis, MN 55455, USA

**Keywords:** cardiac muscle, troponin, myosin, myosin-binding protein c, titin, heart disease, sarcomere

## Abstract

The cardiac sarcomere is a triumph of biological evolution wherein myriad contractile and regulatory proteins assemble into a quasi-crystalline lattice to serve as the central point upon which cardiac muscle contraction occurs. This review focuses on the many signaling components and mechanisms of regulation that impact cardiac sarcomere function. We highlight the roles of the thick and thin filament, both as necessary structural and regulatory building blocks of the sarcomere as well as targets of functionally impactful modifications. Currently, a new focus emerging in the field is inter-myofilament signaling, and we discuss here the important mediators of this mechanism, including myosin-binding protein C and titin. As the understanding of sarcomere signaling advances, so do the methods with which it is studied. This is reviewed here through discussion of recent live muscle systems in which the sarcomere can be studied under intact, physiologically relevant conditions.

## 1. Introduction

### 1.1. The Sarcomere as the Functional Unit of the Muscle Cell

In the cardiac muscle, the sarcomere is the functional unit driving contraction. The sarcomere consists of a pattern of repeating contractile and regulatory proteins organized into thin and thick filaments [1]. The thin filament is where the regulatory unit of contraction is located, and it is complexed together with the myosin-based thick filament, which is where force is generated [1]. These interlacing myofilaments work in strictly timed synchrony to both regulate and generate the forces needed for cardiac muscle contraction. Within the physiological time scale of a single heartbeat, sarcomere activation is controlled through the positioning of the regulatory unit found on the thin filament. This mechanism includes the input of calcium dynamics interacting with the thin filament regulatory proteins, troponin and tropomyosin, as well as the thick filament protein, myosin [1,2]. Additionally, inter-myofilament-signaling proteins, including titin and myosin-binding protein C, play an important role in maintaining proper cardiac sarcomere activation and contraction [3,4]. These finely tuned mechanisms can be greatly altered through inherited mutations in these sarcomeric proteins, thus making understanding the roles of these components in contraction critical to therapeutic design [5,6,7]. This review seeks to highlight recent findings that position the sarcomere as a signaling nexus in cardiac muscle contraction and, thus, an important target in treating heart disease.

### 1.2. Cardiac Muscle Contraction

Overview: Contraction begins in the cardiac muscle through the electrical stimulation of the muscle. This stimulation generates an increase in intracellular calcium levels, first through a small amount of calcium which enters through dihyropyradine receptors (DHPR), L-type voltage-gated calcium channels [8]. The entry of this small amount of calcium leads to a bulk calcium release through ryanodine receptors (RYR) found in close spatial juxtaposition on the membrane of the sarcoplasmic reticulum (SR). This signaling process is known as calcium-induced calcium release (CICR) [9]. Intracellular calcium binds to troponin C (TnC), found on the thin filament, which causes the protein to undergo a global conformation change, becoming more compact [10]. This conformational change strengthens the interaction of TnC and troponin I (TnI) causing a chain reaction in which the inhibitory binding of TnI to actin is simultaneously reduced. With this inhibition removed, tropomyosin (Tm) can now move its position on the thin filament and expose myosin-binding sites on actin [8,11]. Additionally, troponin T (TnT) and Tm interactions cause a cooperative transmission of this sarcomere activation along and across the thin filament through a series of orchestrated protein–protein interactions [1,11,12]. Myosin, now able to bind to the thin filament, can form strong cross-bridges which both helps to stabilize the positioning of the thin filament [1] and leads to the generation of force [9].

For relaxation to occur, calcium needs to be removed from the cytoplasm and stored back in the SR. The key proteins involved in this process are the sarco(endo)plasmic reticulum Ca^2+^-ATPase 2a (SERCA2a) and phospholamban (PLN), both found on the SR membrane [13,14]. This SERCA2a/PLN complex is the driving force for intracellular calcium cycling in cardiac muscle [15]. Through SERCA2a, both the rate of calcium uptake into the SR and SR calcium load are regulated, thus SERCA2a has an important role in both cardiac relaxation and contraction [15,16]. PLN negatively regulates SERCA2a function when dephosphorylated, impacting the SERCA2a function, and serving as a critical target of phosphorylation signaling to affect heart performance [15,17].

## 2. Regulation of Sarcomere Contraction

### 2.1. Thin Filament Regulation of Sarcomere Contraction

**Figure 1:**
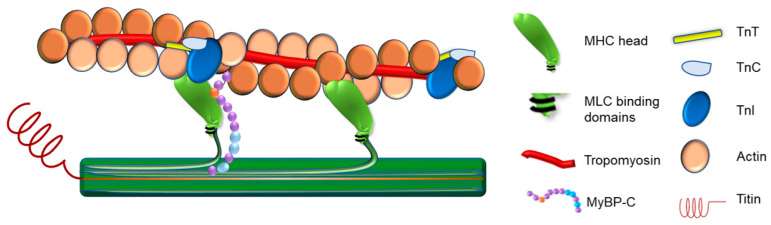
Illustration of the Cardiac Muscle Sarcomere. In this figure, the illustration shows the proteins assembled to construct the cardiac muscle sarcomere.

The cardiac thin filament regulatory complex is a highly developed multiprotein system composed of seven actin monomers, one troponin complex (cardiac troponin I ‘TnI’: cardiac troponin T ‘TnT’: cardiac troponin C ‘TnC’), and one Tropomyosin (Tm) coiled-coil dimer [18] (Figure 1). This section will examine the regulation of cardiac sarcomere contraction at the level of the thin filament.

#### 2.1.1. TnC-Calcium

Calcium plays an essential role in cardiac muscle contraction at the level of the sarcomere through its interaction with the troponin regulatory complex, specifically binding to TnC [2,19]. *TNNC1* encodes the cardiac TnC isoform that is also expressed in slow skeletal muscle. There have been no reports of troponin C isoform transitions during myocardial development [17,20].

TnC is a dumbbell-shaped molecule with two globular domains (the N and the C domains) connected by central linker [21,22]. TnC belongs to E–F hand-type Ca^2+^-binding protein family. TnC has four E–F hand motifs, two in each N and C domain. The key domain involved in Ca^2+^ regulation of cardiac muscle contraction is the Ca^2+^-binding site II in the N-domain of TnC [1]. This has led to the N-terminal domain being referred to as the regulatory domain and the C-terminal being considered the structural domain. As previously mentioned, calcium binding to TnC results in a conformational change in the structure of the protein, leading to TnC becoming more compact [10,17]. These changes provide a mechanism for TnC, in the calcium-bound state, to interact with TnI, leading to sarcomere activation [19,23].

#### 2.1.2. TnC-TnI

TnI is known as the inhibitory subunit of the troponin complex due to its demonstrated role of inhibiting cardiac muscle contraction through strong actin binding at low calcium concentrations [17]. Within TnI, there are at least two actin-interacting sites: the inhibitory region and an additional actin-Tm binding site. The inhibitory region is enriched with basic amino acids, and this sequence is highly conserved among species [2,24]. The inhibitory region of TnI is critical in regulating myofilament activation/inactivation [2]. Once calcium binds to TnC, TnI is able to bind to the now exposed TnC hydrophobic patch found on the N-terminal region [2,23]. The portion of TnI that is involved in this mechanism is referred to as the switch region and this binding between TnI and TnC helps to remove the inhibitory arm of TnI from the actin-Tm interface exposing myosin binding sites found on actin [2,17,19]. This interaction is crucial to the development of force in cardiac muscle and even single amino acid changes can lead to impaired function [23,25,26,27]. Additionally, the mechanism is highly tunable through post-translational modifications such as phosphorylation changes, which will be discussed below.

#### 2.1.3. TnT-Tm

TnT is able to bind and interact with the troponin complex, Tm, and actin. TnT is a polar molecule with 30% acidic and 20% basic residues in its amino acid composition [17]. Mild chymotryptic treatment yields two soluble fragments of TnT, namely T1 and T2. The T1 segment is the N-terminal portion and binds to Tm strongly in the region where TnT is situated closest to the Z-disk extending towards the head–tail interaction site of Tm. The T1 segment also plays a crucial role in the activation of the thin filament by regulating the Tm–Tm interaction in the head-to-tail linkage of the Tm strand [27]. The C-terminal region of TnT (T2), is about 100 residues, and forms the globular domain of the Tn complex and binds both TnI and TnC [17,27,28,29]. TnT plays a major role in the cooperativity of activation of the sarcomere [30]. In the absence of calcium, the N-terminal domain of TnT has been shown to maintain the inhibition of myosin binding by stabilizing the Tm position on actin [2]. When calcium is present and bound to TnC, conformational changes in TnI are propagated from the troponin complex to Tm through TnT [2,23]. 

Thin filament activation cooperativity is through the interaction of the troponin complex, Tm, and actin [2]. The thin filament is stabilized by Tm molecules [31], wherein Tm is formed as a homodimer or heterodimer that is linked end-to-end to form continuous α-helical coiled-coils that follow the F-actin helix, where they, in turn, control muscle contraction by blocking the myosin target sites on the thin filament [32]. The positioning of the Tm protein is part of what determines if the thin filament is in the blocked, closed, or open state as it is mainly responsible for blocking the myosin binding sites on actin [2,17]. Importantly, previous work has demonstrated that changes in the troponin complex driven by calcium binding impact Tm through the highly coordinated conformational changes as discussed above [17]. These changes allow for strong myosin cross bridges to form, which also aid in the shift of Tm positioning on actin, thus exposing more actin binding sites [2,33]. Although these interactions may start off locally, as they progress throughout regions of the thin filament they lead to an additive, cooperative activation of the full system.

### 2.2. Thick Filament Regulation of Sarcomere Contraction

Striated muscle generates contractile force by the interaction of actin and myosin, leading to a shortening of the sarcomeres in a load-dependent manner [34,35]. In addition to the mechanism mainly initiated in the thin filament, as discussed above, sarcomere control mediated by thick filament regulation has more recently become an area of focus [36,37,38]. This includes emerging evidence that implicates thick filament-based regulation as a key component in sarcomere signaling. This section will review the regulation of muscle contraction moderated mainly by the thick filament of the sarcomere and its major component proteins. 

The thick filament is composed of the protein myosin, a large, hexameric, motor protein that occupies the center of the sarcomere where it partially overlaps with thin filaments, through inter-myofilament interdigitating, and slides past actin filaments in an ATP-dependent manner to mediate muscle contraction [34,39] (Figure 1). Hexameric myosin (520 kDa) consists of two myosin heavy-chain (MHC) subunits and four myosin light-chain (MLC) subunits (two essential and two regulatory light chains) [39,40,41]. Each MHC folds upon itself at the N-terminus, forming a globular head region (subfragment-1), so that each myosin molecule has two globular heads. These head regions are referred to as the motor region of the molecule as they contain binding sites for actin, ATP, MLCs, and divalent cations [39]. The central segment of the head region of the myosin protein contains an actin-binding site and an ATP-binding pocket which is located at the attachment site within the N- terminal segment. Both the central and the N- terminal segments of myosin together form the motor domain. The C-terminal portion of the head region of myosin forms the regulatory domain [42], where one regulatory light chain and one essential light chain bind [43], and this domain links the motor domain to the myosin molecule tail [43]. The MHCs fold together at their C-terminus forming the tail region, which is a dimerized coiled-coil α-helix. This region anchors and positions the myosin motor domains to interact with actin. It also contains the binding sites for myosin assembly into the sarcomere [44]. 

An active myosin cycling state is where a rapid ATP turnover is observed and involves ATP hydrolysis activated by actin. At low [Ca^2+^], cross-bridges cycle between detached and weakly bound states, with ATPase in myosin partially active. In high [Ca^2+^], cross-bridges can enter the strongly bound state, and myosin ATPase becomes fully active [29]. This Ca^2+^-thin filament paradigm emerged around the 1970s [29], but it was later apparent that other pathways are involved to address metabolic efficiency [45]. Specifically, the different functional states in the thick filament of the muscle sarcomere were discovered and the super-relaxed state (SRX) was described by monitoring the kinetics of a fluorescent ATP analog (mantATP) in permeabilized rabbit skeletal muscle fiber in 2010 [38]. After this breakthrough, a series of studies established different states of myosin in striated muscles [46,47]. The second state is the disordered–relaxed state (DRX) with partial myosin ATPase activity with no or partial actin activation. This state is represented by the cross-bridges protruding out from the myosin backbone into the inter-myofilament space but largely restricted from binding to actin by regulatory proteins such as troponin and tropomyosin. The super-relaxed state (SRX) is marked by cross-bridges trapped in the myosin backbone with inactive myosin ATPase (ATP turnover time >100 s) [38]. Due to the lower ATP turnover rate, the SRX is characterized by an energy-saving state which modulates energy utilization, especially in cardiac muscle [36,37]. 

According to the thick filament regulation paradigm, the availability of myosin for actin binding, determined by the location of myosin relative to the thin filament, is the major determinant of the functional states in the muscle [45]. Trivedi et al.’s human β-cardiac myosin model illustrates a subfragment (S1) that incorporates ATP and actin-binding sites connected to an α-helix of the myosin-heavy chain, which binds the ELC and RLC [48]. A three-dimensional structure assay revealed that the RLC stabilizes the α-helical neck region of the myosin head [43]. Once phosphorylated, the RLC enhances the mobility of myosin cross-bridges through charge repulsion and functions as a ‘lever arm’ to protrude toward actin thin filaments in cardiac fibers [49,50]. Cardiac and skeletal RLC contain a serine residue which can be phosphorylated by myosin light-chain kinase (MLCK) at submaximal [Ca^2+^]. Specifically, MLCK forms a Ca^2+^-calmodulin-MLCK complex, and switches the RLC to the force-generating state by myosin head arrangement [49,50,51]. 

Historically, Ranke initially reported that repetitive skeletal muscle stimuli led to greater twitch contraction than the initial twitch [52]. Furthermore, the RLC of vertebrate striated muscle was shown to be phosphorylatable [53]. Thereafter, there has been a great interest in the isometric twitch potentiation during trains of stimuli at low frequency (staircase) or after a tetanic stimulus (post-tetanic potentiation; 100–150 Hz, ~2 s) mostly in the fast-twitch muscles [54]. In a more recent study, the use of genetic ablation of skeletal muscle MLCK (skMLCK) in mice showed that tetanic stimulation in the extensor digitorum longus did not lead to RLC phosphorylation and isometric twitch force potentiation, in contrast with data from wildtype mice [54]. Interestingly, staircase potentiation (10 Hz, 15 s) was observed in skMLCK knockout muscle, but it was about 50% lower than in the wildtype. Conversely, the overexpression of skMLCK enhanced RLC phosphorylation as well as staircase potentiation [55]. 

The relatively reduced RLC phosphorylation present in the slow-twitch muscle is attributed to the lower skMLCK activity and higher myosin light chain phosphatase (skMLCP) activity, as well as calmodulin [56]. However, primarily slow-twitch muscles such as mouse soleus do not show any potentiation by staircase or post-tetanic potentiation, even with increases in RLC phosphorylation [57]. This shows the potential involvement of isoform differences in the proteins of the contractile apparatus such as the RLC, light-chain-binding domain, or S-1 [57].

Contraction-induced post-translational modification of myosin by MLCK was found to be a main mechanism for functional potentiation in cardiac muscle [58]. This mechanism of functional potentiation through the enhancement of Ca^2+^ sensitivity through the activation of MLCK and the resulting translocation of the phosphate group from ATP to a serine residue of RLC is also found in skeletal muscle [55]. Specifically, multiple investigations of the phosphorylation of RLC in permeabilized skeletal muscle fibers observed the force-pCa relationship curve shifting to the left, denoting increased Ca^2+^ sensitivity of contraction [49,50,59]. Also, phosphorylation of RLC by repeated activation in intermediate Ca^2+^ concentrations (pCa 5.5–6.2) in permeabilized murine skeletal muscle fibers increased the rate constant of force redevelopment (K_tr_), but not at maximal [Ca^2+^] [49]. Furthermore, thin filament cooperativity was found to be required for the force potentiation by RLC phosphorylation at submaximal [Ca^2+^], as the partial extraction of TnC prevented the increase in calcium sensitivity of steady-state force development [49]. These observations show the involvement of cross-bridge binding in the enhancement of Ca^2+^ activation in functional potentiation. Additionally, an increase in Ca^2+^ sensitivity showed a positive relationship with actomyosin ATPase, suggesting an increase in the number of cross-bridges poised to interact with the thin filament, rather than altering the force per cross-bridge [50]. From a structural point of view, phosphorylation of RLC disrupts the ionic interactions between myosin heads and the thick filament, leading to the increased mobility of myosin cross-bridges [55]. 

Interestingly, cardiac RLC has been found to be dephosphorylated approximately 100 times slower than skeletal muscle RLC and with noted functional relevance [60,61]. This feature is in contrast to the MLCK in the smooth muscle and skeletal muscle [61]. Recently, the fourth isoform of MLCK (MLCK4) was discovered in the cardiac muscle [62]. In addition to other MLCK isoforms that are found in smooth muscles (MLCK1), skeletal muscles (MLCK2), and previously probed in cardiac muscles (MLCK3), MLCK4 contains intrinsically low constitutive activity that is independent from Ca^2+^-calmodulin, and this contributes to the maintenance of RLC phosphorylation in cardiac muscle. In the relaxed striated muscle sarcomere, a portion of RLC molecules remain unphosphorylated so that a sub-population of myosin heads is kept locked in the filament backbone. This state is referred to as the interacting heads motif (IHM) state [63,64]. The above description of the mechanism of phosphorylation of RLC suggests a close relationship between the functional state of myosin in equilibrium between SRX and DRX, with RLC phosphorylation status determining the conformation of myosin heads [65]. Furthermore, a decrease in RLC phosphorylation was observed in both heart failure patients, impacting the myosin light chain 2 isoform, [66,67] as well as in animal models [47,68,69,70,71]. 

### 2.3. Inter-Myofilament Signaling in the Sarcomere

**Figure 2:**
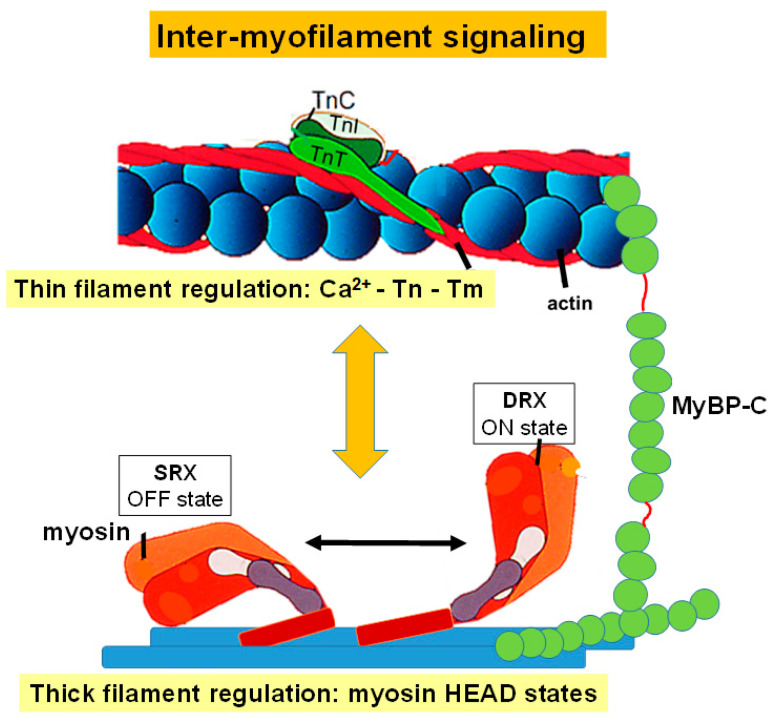
Inter-myofilament Signaling in the Cardiac Sarcomere. This figure depicts regulation of thin filament activation, which is driven through calcium binding to troponin C (TnC), TnC and troponin I (TnI) interactions, and the position of tropomyosin (Tm). It also illustrates activation of the thick filament regulated through myosin head states. Myosin-binding protein C has also been included due to its role in regulating interactions between the thin and thick filament.

As has been discussed above, regulation of contraction of via the cardiac sarcomere is a highly tuned process involving mechanisms specific to the thin filament or the thick filament. As the activation or inhibition of one filament system clearly influences the positioning or status of the other, this leads to the assumption that there are important aspects of inter-myofilament signaling governing overall sarcomere performance. This signaling is not only driven between the thin and thick filaments but also through proteins that are able to interact with both myofilament components (Figure 2). 

#### 2.3.1. Myosin-Binding Protein C

Myosin-binding protein C (MyBP-C) is an ~140 kDa sarcomeric accessory protein [72] that was isolated in 1971 by Starr and Offer as impurities contaminating crude skeletal muscle preparations [73]. In humans, cardiac MyBP-C is encoded by the MYBPC3 gene [74]. Cardiac MyBP-C is located in the C-zone of each half A-band of the sarcomere (Figure 1) and primarily has eight globular immunoglobulin-like domains and three fibronectin type-III domains referred to as C0-C10 from the N-terminus [4,74]. Cardiac MyBP-C has two linker domains: A proline alanine-rich domain between the first two Ig-like domains (C0 and C1), and an MBPC3 motif (M-domain) between the second and third Ig-like domains C1 and C2 [4,74]. The C5 Ig-like domain has a 28 amino acid loop that is also unique to the cardiac isoform [75]. In addition to the crucial role of cardiac MyBP-C in the organized assembly of actin and myosin filaments into sarcomeres [76,77], it has mechanical, signaling, and transport functions as well as a regulatory role in sarcomere contraction [78,79].

The sarcomeric location of MyBP-C and its ability to interact with myosin through the C-terminal domain put the protein in a prime position to influence the state of myosin and thus the development of force [4]. Previous work has demonstrated an interaction between the N-terminal domain of MyBP-C and myosin, with the C0–C2 domains playing an important role in the modulation of the thick filament protein [78,79,80,81]. Much of this interaction is driven by the phosphorylation of MyBP-C which will be discussed later in this review. The ability of MyBP-C to bind myosin suggested that it may be able to hinder myosin head movement, specifically through maintaining myosin in the recently described SRX state [4,38,81,82,83] where the number of force-generating heads is reduced. This is in line with experimentation, demonstrating that the phosphorylation of MyBP-C leads to a greater number of myosin heads in the active DRX state and a reduction of myosin heads in the SRX state [82,84]. Together this provides strong evidence of a role for MyBP-C in regulating muscle contraction through thick filament interactions.

MyBP-C is also able to influence muscle contraction through interactions with the thin filament. Shortly after it was shown that MyBP-C binds myosin, work also demonstrated its ability to bind to actin [85]. Extensive research has been done to better understand the role MyBP-C plays in regulating contraction through the thin filament [4,81] and this has shown, again, it is through the N-terminal domain of MyBP-C that the thin filament can be activated. This is an opposite result to that seen in the thick filament. This level of activation is similar to that driven by an increase in calcium or by the presence of strong myosin cross-bridge binding. Although this may seem counter-intuitive, EM work by Mun et al. [86] showed that the N-terminal of MyBP-C is able to shift the position of Tm to the “open” state, leading to increased thin filament activation even at low levels of calcium. The ability of MyBP-C to interact with both the thin and thick filament, resulting in opposing outcomes on force production, demonstrates the highly complex and critical role of MyBP-C in the regulation of contraction.

#### 2.3.2. Titin

Titin is a giant protein, ~3MDa, that links the Z-discs to the M-lines in the middle of the sarcomere, acting as a scaffold, and is thought to be responsible for the passive tension within the sarcomere [87] (Figure 1). The titin gene is enormous comprising 363 exons. Alternative splicing, allowing for isoform switching, and post-translational modifications are the main mechanisms for the long-term modulation of passive tension in the heart [87,88]. Over the I-band of the sarcomere, titin is extendable and contains immunoglobulin-like domains arranged in tandem, including the Proline, Glutamate, Valine, and Lysine-rich (PEVK) region and the N2B element, all functioning in series as distinct spring elements [89]. Over the A-band, the titin fragment is fixed and is comprised of regular Immunoglobulin and fibronectin type 3 domains that form super-repeats [89]. The titin C-terminus, which contains a kinase domain, is positioned at the M-band, whereas the N-terminus localizes by the Z-disk of the sarcomere. Titin filaments overlap and interconnect at both the Z-disk, from adjacent sarcomeres, and M-band, from the opposing half sarcomere, forming a continuous filament alongside the myofibril. 

Single-molecule studies revealed that titin resembles a “modular polymer” of connected, distinctly extensible segments, that respond sequentially to the tension applied in the axial direction [89]. Each titin domain possesses intrinsic folding properties that govern the unfolding/refolding order [90]. At baseline, in absence of an external force, titin acts as a series of individual, interconnected springs, wherein each unit/fold of the spring is bent over, and resists stretch. When an external force is applied, titin domains unfold and contribute to the “entropic” elasticity of the newly extended spring that is longer and has a new spring constant. When the external force is relieved, each unit of titin refolds individually, going back to its baseline status [87].

A major role of titin in muscle contraction is the contribution of passive force, which helps to stabilize sarcomeres and is driven through the structural components of the protein during muscle stretch [91,92]. Previous work has demonstrated that when a muscle is relaxed, segments of the titin protein are tightly coiled or contracted. As the muscle is stretched, the components of the titin protein extend and passive force develops in direct resistance to stretch [93]. Titin is bound to both myosin and actin, acting as a bridge between the thin and thick filaments [92,94]. One way that the passive stiffness of titin can be modulated may be through interactions between titin and actin in the thin filament. Previous studies have shown that in cardiac muscles, the PEVK-region of titin is able to slow thin filament sliding which demonstrates these interactions contribute significantly to the development of passive tension [3]. This effect can also be tuned by calcium levels, specifically in the presence of cardiac-expressed EF-hand protein S100A1, where changes in intracellular calcium concentrations during the physiological timescale of a heartbeat can impact the interactions of actin and PEVK [3].

Titin, specifically the stiffness of the protein, is also involved in muscle contraction through the mechanism of length-dependent activation. In animal models that have very compliant forms of titin there is a corresponding reduction in length-dependent activation, with both myosin and troponin positioning being affected, suggesting titin strain plays an essential role [95]. A second proposed model for length-dependent activation involving titin includes the previously discussed inter-myofilament protein MyBP-C. In this model, it is proposed that stretch in cardiac muscle during diastole results in titin strain that is sensed by the thick filament. The thick filament is then able to transmit this strain to the thin filament through MyBP-C leading to length-dependent activation [95]. 

### 2.4. Post-Translational Modifications in Sarcomere Signaling

Many sarcomeric proteins involved in the regulation and tuning of cardiac muscle contraction are targets of post-translational modification (PTM). This section will more closely examine how these modifications impact and modulate sarcomere protein function, sarcomere activation, and force development.

#### 2.4.1. Troponin

There are multiple sites for PTM among members of the thin filament troponin regulatory complex [6,29,96,97]. When focusing on TnC, previous work has identified regions within the protein that are targets for a variety of PTMs including acetylation, glycation, S-nitrosylation, and phosphorylation [96,97]. The majority of these modifications impact calcium binding to TnC with acetylation leading to an increased calcium affinity of TnC [98], glycation impacting calcium sensitivity in diseases such as diabetes [99], and S-nitrosylation causing a desensitization to calcium and thus impacting myosin cross-bridge rates which can lead to a decrease in cardiac contraction [100]. Phosphorylation of TnC is also important in tuning calcium sensitivity of the thin filament [98,101] and although phosphorylation targets have been identified, S89 and S98 [97], it is still unclear what the direct physiological role this PTM has on TnC. 

The impact of phosphorylation on protein function in the physiological context has been studied much more extensively with regard to TnI and TnT. Phosphorylation sites have been found on both TnI and TnT with specific sites, such as serine and threonine, and phosphorylation pathways, including through PKA, PKC, and the Rho-A/ROCK pathway, resulting in varying outcomes [6,29,102,103,104,105]. For TnI, PKC phosphorylation within the inhibitory domain can lead to either an increase in calcium sensitivity in the myofilaments and thus an increase in cross-bridge cycling or a reduction in both calcium sensitivity and cross-bridge cycling [102]. Physiologically, PKA phosphorylation at TnI serine 23/24 has a key role in muscle relaxation, as shown in adult cardiac myocytes [105]. Specifically, previous work using gene transfer and transgene expression in intact cardiac myocytes demonstrated when a non-PKA phosphorylatable TnI is expressed, there is a prolongation of relaxation. Using the same experimental paradigm, the expression of a PKA phosphomimetic TnI at serine 23/24 leads to a significant acceleration in myocyte relaxation kinetics [105]. There have been a few phosphorylation targets identified in TnT with one, Thr-206, seeming to have the most impact on muscle function [6]. Phosphorylation of this site has been shown to reduce calcium sensitivity and thus reduce force development. From these previous studies, there is no question that PTM plays a meaningful role in the modulation of muscle contraction through the troponin complex.

#### 2.4.2. MyBP-C

The impact that PTM has on MyBP-C, specifically phosphorylation, has been extensively studied [4,106]. With multiple phosphorylation targets and mechanisms, it is widely accepted that MyBP-C is phosphorylated at a physiological baseline, which has an impact on the role of MyBP-C in regulating muscle contraction [4,106,107,108]. When MyBP-C is unphosphorylated, as shown in previous studies [107,108], the contractility of the heart is decreased and hypertrophy develops. An apparent decrease in the phosphorylation levels of MyBP-C is also found in a variety of other models of cardiac dysfunction as well as in human patients [106]. Previous work has shown that the sites of MyBP-C that are phosphorylated can impact myofilament structure and function. This includes the positioning and resulting state of myosin heads, actin-myosin interactions, and rates of cross-bridge cycling [75,109]. 

It should be noted that while phosphorylation of MyBP-C has been the area of greatest focus, MyBP-C can also be modified through other PTM mechanisms. Redox modifications have been shown to target MyBP-C for degradation in cases of cardiotoxicity [110], leading to a reduction in calcium sensitivity and resulting decrease in force production [100], and reduced cross-bridge cycling [111]. MyBP-C can also be acetylated, which may lead to cleavage of the protein and heart failure [112]. Together this makes MyBP-C a critical target for PTM in regard to cardiac function. 

#### 2.4.3. Titin

The inter-myofilament protein titin is also a target of PTM through phosphorylation. Due to the large size of the protein many potential phosphorylation sites have been predicted, as previously reviewed in the works cited in this section. However, only a few sites have been thoroughly studied, specifically, those found in the PEVK and N2B regions [3,113]. It has been shown that phosphorylation of the titin N2B domain leads to a reduction in passive tension through a reduction in titin stiffness [3,114,115]. Interestingly, previous studies have also shown that phosphorylation of the titin PEVK domain results in an increase in titin stiffness and an elevation of passive tension in cardiac myocytes [116]. This points to an important location-specific effect of titin phosphorylation on titin function. 

## 3. Biosensing in the Sarcomere

As has been discussed here, there are enumerable sarcomeric proteins that are critical to the proper development of force within the cardiac muscle. This understanding is based on many previously established methods to study changes in intracellular calcium kinetics and protein interactions within the heart but it has been more difficult to make these inquiries in the physiological setting of an intact muscle [8,117]. This section will explore some recent advances in the field towards this end.

### 3.1. Cardiac TnI A164H Histidine Button

In order to address the health issue of ischemic heart disease, in which heart muscle dysfunction is due to a reduction in myofilament calcium sensitivity driven through acidosis, previous works have focused on the thin filament regulatory protein TnI [118]. This research highlighted the TnI isoform-specific protection against ischemic conditions, by capitalizing on the knowledge that the fetal TnI isoform, ssTnI, prevents these negative effects on contractility and that this protection is derived from the C-terminus of TnI, specifically at a single histidine in ssTnI [6,119]. This led to the replacement of alanine with histidine at position 164 (A164H) in TnI, mimicking the histidine found in fetal ssTnI, in isolated adult cardiac myocytes and whole hearts from mice as well as through gene transfer in human myocytes from failing hearts. The expression of this “histidine button” led to enhanced diastolic performance and pressure development during acidosis and ischemia-reperfusion, as well as increased contractile function in the human myocytes from failing hearts [118]. These changes were predominately seen during acidosis, hypoxia, and ischemia, suggesting that the histidine substitution acts as a “pH-switch”. Based on the atomic structure of TnI [120], this would result in TnI being in a conformation that most favors binding to TnC, leading to increased contractility even in these highly acidic conditions. Together, along with work that elucidated the molecular mechanism of the histidine modification [121] and the high degree of evolutionary conservation of these TnI residues [122], this work provides an example of a thin filament protein being able to sense changes in the intracellular milieu in an intact system and positively respond as the environment moves from physiological baseline to disease conditions [118].

### 3.2. Sarcometer

**Figure 3:**
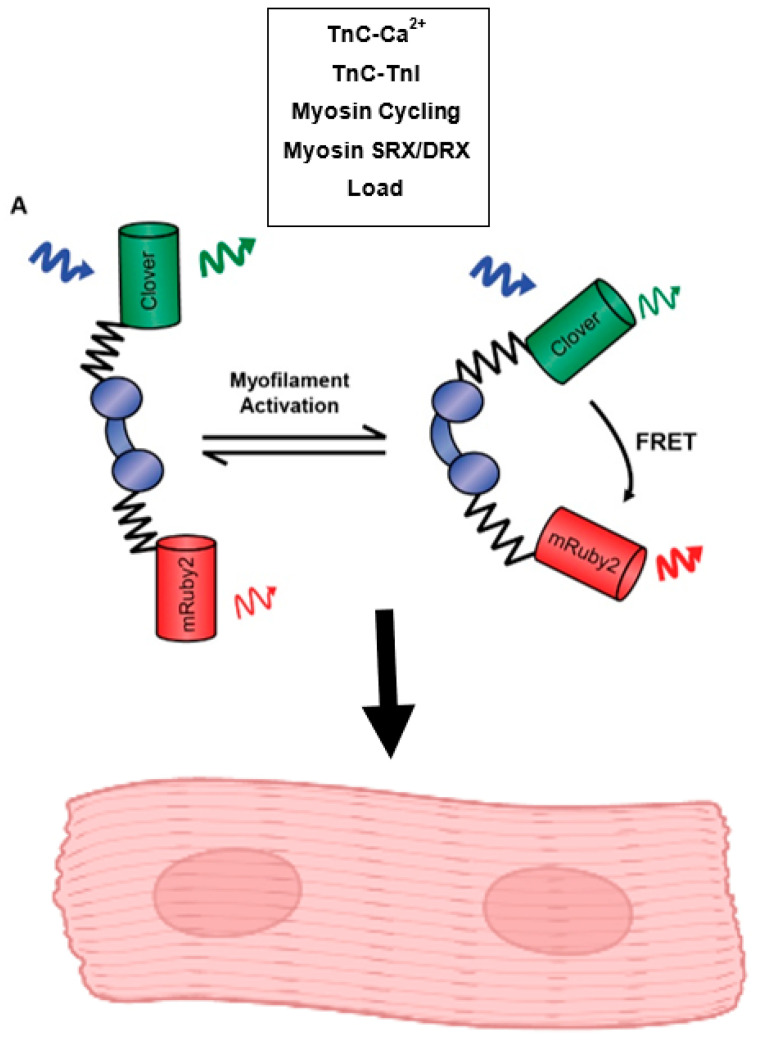
Function of the Cardiac-Expressed Sarcometer. This model illustrates the construction of the cardiac TnC biosensor which functions through changes in FRET fluorescence driven by the conformational changes TnC undergoes in response to calcium binding, TnC-TnI interactions, myosin cycling, the activation states of myosin, and changes in load. This biosensor has been expressed in a stable transgenic mouse line and can be used to study sarcomere activation in isolated, intact cardiac myocytes and intact cardiac papillary muscles.

The examination of sarcomeric activation at the level of the thin filament in real time in intact muscle has been a long-sought goal of the field. To this end, recent work has resulted in the development of a cardiac-specific FRET-based biosensor engineered into cardiac TnC and expressed in myofilaments in a transgenic mouse line [123]. This mouse model allows for the reporting of the effects of many known thin filament ligands, including calcium, TnI, Tm, and myosin cycling, in the time scale of a single twitch under physiological conditions in intact muscle (Figure 3). Results from this system have provided evidence relating to the conformational changes TnC undergoes upon calcium binding, and how this relates to the ability of TnC to act as a nexus point for sarcomeric signaling, many pathways of which have been previously highlighted in this review. Specifically, using small molecules or genetic models to perturb the ability of TnC to interact with known ligands, such as TnI, demonstrated the ability of the biosensor to detect the activating effects of the TnI switch domain binding to TnC, a thin filament-driven pathway of contraction regulation. It was shown that the biosensor is able to sense the state changes of the TnI switch region and it is widely accepted that the switching mechanism of TnI directly affects the positioning of Tm on the thin filament. Therefore, this can be used as a proxy for the localization of Tm during the time course of a twitch contraction as well. The use of small molecules also illustrated the ability of the biosensor to sense changes in myosin cross-bridge rates, a thick filament-driven pathway of contraction regulation. Importantly, the biosensor reported changes in myosin states in a load-dependent manner, providing support for the ability of myosin cross-bridge formation to influence thin filament activation in the physiological environment of an intact, loaded cardiac muscle [123]. This is in line with myosin having a critical role in maintaining sarcomere activation versus initiating activation. With the design of this biosensor, there is now the opportunity to expand understanding of the many mechanisms which regulate and tune heart performance, especially in the context of sarcomere dysfunction in cardiac disease with the potential to leverage this system in order to identify potential new therapeutics.

## 4. Mechanosensing and Sarcomere Activation

Myocardial force produced by the myofilament can be sensed by components within the sarcomere. More specifically, it is thought that this process involves mechanosensory proteins within the sarcomere that are capable of detecting and responding to mechanical load. This section will focus on work that has been done to elucidate sarcomeric mechanosensors and how these sensors advance our understanding of sarcomere activation and force generation.

A well-described mechanosensory protein in the sarcomere is myosin [124]. As myosin is not only sensing force but also plays a key role in generating force, the protein must be precisely optimized both mechanically and kinetically. This ensures myosin’s ability to properly respond to the development of and changes in force. It is thought that mechanical force, as in active contraction, is able to alter myosin cross-bridge cycling rates in a manner that is isoform-specific, with cardiac myosin being impacted by load through the slowing of the rate of myosin detachment from actin [124]. Previous studies have also suggested that the mechanosensing mechanism of myosin is connected to the physical state of myosin [125]. Prior to contraction, many myosin motors are aligned closely to the surface of the thick filament. During a twitch, a portion of these motors transition out of this resting state, the number of which are dependent on loading conditions. This load-dependent process creates a direct feedback mechanism between the myosin state and muscle active loading [125,126]. Together, this work demonstrates the important role of myosin in mechanosensing in the sarcomere.

Titin has also been described as a necessary sarcomeric mechanosensor, specifically in relation to length-dependent activation (LDA) of the sarcomere [87]. As this was previously discussed above, it will only be briefly touched upon here. Titin has been shown to be able to interact with both the thin and thick filament, making it an attractive candidate for mechanosensing. Previous work has demonstrated that changes in passive tension as driven through titin can significantly reduce LDA [95]. Additionally, the ability of titin to participate in inter-myofilament signaling between the thin and thick filament may allow it to act as a link to transmit mechanical information relating to thin filament activation or myosin cross-bridge kinetics between these two important sarcomere components. There is also evidence for a role for other sarcomeric proteins or lattice spacing in LDA which the following reviews have extensively covered [127,128,129]. Within muscle, titin has been proposed to be a “strain-sensor” with previous research demonstrating that mechanical stress within the physiologic range of force is sufficient to activate the titin kinase domain [130,131]. Once active, the kinase domain is able to interact with multiple signaling pathways that are important for muscle maintenance, which may lead to functional and structural changes in the muscle in response to mechanical force [131].

There are also important structural elements that play a role in mechanosensing, specifically those proteins that make up the cytoskeleton. In striated muscle sarcomeres, the contractile proteins actin and myosin are organized by a network of specialized proteins which combine structural and signaling functions [130,132,133]. The cytoskeleton represents the main structural scaffold essential in the regulation of mechanical resistance, morphological integrity, and cell shape, providing the uniform transmission of tension along myofibrils. Moreover, the cytoskeleton allows an integrative connection to the extracellular matrix and therefore enables bidirectional signaling, allowing the muscle to sense and rapidly respond to mechanical stimuli through connections with the cytoskeleton and activation of signaling cascades [134]. The cardiac muscle cytoskeleton is complex, composed of diverse macromolecular assemblies and yet intricately organized to coordinate the network between the sarcomere and cytoskeleton essential for muscle contraction [135]. 

The cardiac muscle cytoskeleton contains numerous proteins including actin, tubulin, and desmin. These provide a structural lattice to hold myofibrils in place and serve as a scaffold that connects the sarcomere to other organelles, such as the mitochondria and nucleus, which helps preserve cellular integrity and contributes to mechano-transduction [136,137]. The cytoskeleton also includes the Z-disc, which transversely defines the lateral borders of striated muscle sarcomeres and is involved in the mechanical linkage of thin filaments, the uniform transmission of force, and connection of myofibrils to the sarcolemma through interactions with dystroglycan complex. In the Z-disc, cytoskeleton actin filaments are crosslinked by α-actinin. As an integral Z-disc protein, α-actinin has several binding partners with each interaction having a distinct role in the production of contractile action, including muscle LIM protein (MLP), actinin-associated LIM protein (ALP), myopalladin, myopodin, CapZ, cypher/oracle/ZASP, filamin, α-actinin, telethonin-binding protein at the Z-disc (FATZ), and myotilin [136,138,139]. The Z-disc also serves as an anchor site for titin and nebulin/nebulette filament systems, making it crucial for the transmission of contractile force.

Important within the cytoskeleton are structures known as costameres [140]. Costameres are necessary for force transmission from the sarcolemma to the extracellular matrix of the cell. Costameres are located at the Z-line of the sarcomere and are composed of protein complexes, with the two major being the dystrophin–glycoprotein complex and the integrin-vinculin-talin complex [140]. In addition to the structural role costameres play, they also function in cardiac muscle to transduce intracellular signals to the extracellular network and integrate extracellular signals to the intracellular network [140]. This provides a direct system of mechano-transduction via connecting the sarcomere and the extracellular matrix.

## 5. Sarcomere Signaling in Disease and Current Therapeutics 

### 5.1. Sarcomeric Cardiomyopathies

Sarcomeric cardiomyopathies encompass a set of diseases caused by mutations in myofilament proteins [141]. In most cases, single point mutations, in a single gene result in altered contraction and relaxation of the heart that leads to the disease phenotype. Hypertrophic cardiomyopathy (HCM), Restrictive cardiomyopathy (RCM), and Dilated cardiomyopathy (DCM) can all be a result of sarcomeric gene mutations [142]. 

#### 5.1.1. Hypertrophic Cardiomyopathy

HCM is the most common genetic disorder of the heart, with estimates of 1:200 prevalence [143]. HCM was originally diagnosed by unexplained increased left ventricle septal and free wall thickness. In the early 1990′s, HCM was discovered to be a genetic disease of the sarcomere, with mutations being passed down in an autosomal dominate manner [144,145]. Greater than 60% of HCM patients have a disease-causing mutation within a sarcomeric protein [146]. HCM is a heterogeneous disease ranging from severe heart dysfunction leading to sudden cardiac death to mild diastolic dysfunction and normal life expectancy [147]. HCM is the leading cause of sudden cardiac death in young people [141,148]. HCM disease penetrance and heterogeneity may be due to the mutation location itself but in some instances, the same genetic mutation in the same family can lead to different disease outcomes suggesting that epigenetics, environment, or modifier genes could play a role [149,150]. Clinical disease manifestations include left ventricle/septal hypertrophy, diastolic dysfunction, decreased left ventricular chamber volume, myocyte disarray, and fibrosis [141,147,151]. While the human disease progression is incompletely understood basic science has revealed the mechanisms of action for the mutations which cause HCM.

HCM-causing mutations are found in cardiac isoforms of myosin (MYH6/7/8), MyBP-C (MYBPC3), TnI (TNNI3), TnT (TNNT2), TnC (TNNC1), actin (ACTC1), Tm (TPM1), and MLC (MYL2) [151,152,153]. There are more than 1400 known mutations linked to HCM in these genes with myosin, MyBP-C, TnI, and TnT accounting for the majority of mutations [146]. In general, at the biophysical level, HCM mutations result in increased calcium sensitivity of tension or ATPase activity [154,155,156,157,158]. This results, in general, in increased contractility and slower relaxation in cardiac myocytes [159,160,161]. This increase in calcium sensitivity can result in calcium buffering by troponin which is thought to cause slow relaxation but also can lead to arrhythmias that result in sudden cardiac death [162,163]. Although a plethora of mutations leads to common phenotypes, there are different mechanisms to disease pathogenesis. For thin filament mutations, most alter protein–protein interactions in a way that moves Tm sliding to a more closed state allowing myosin access with less calcium [155,164,165]. This can be through directly altering the calcium affinity of TnC or Tm positioning. For myosin mutations, most result in an increase in the DRX state thereby increasing the number of myosin motors in the on state which increases force and slows relaxation [48,166]. The increased number of myosin heads binding to actin can lead to increased calcium sensitivity of the thin filament as well. MyBP-C is the only sarcomeric protein with the majority of mutations being truncation mutations. These typically don’t result in a poison peptide like the other sarcomeric proteins but result in haploinsufficiency [167]. This has been shown to result in increased calcium sensitivity and increases in the DRX state of myosin [167]. Overall, these mutations result in an overactive sarcomere which results in slow relaxation and increased contraction that can lead to maladaptive hypertrophy. 

#### 5.1.2. Restrictive Cardiomyopathy

RCM is characterized by normal wall thickness with increased stiffness of the heart and major diastolic dysfunction [168]. It is relatively rare and usually results in heart failure with transplantation at a young age being common. Inherited RCM due to mutations in the sarcomere is an autosomal dominate disorder. Mutations in the same sarcomeric genes found in HCM are also reported in RCM including TnI, myosin, TnT, actin, Tm, and MLC [154,168]. Several studies have shown an overlap between HCM and RCM, both clinically and biophysically. Clinically it was found that even a specific mutation that causes HCM can cause RCM in the same family [149,150]. In addition, the same mutation was found in two separate families with one having HCM and another having RCM [169]. At the biophysical level RCM mutations tend to result in increased calcium sensitivity of tension or ATPase activity, similar to HCM mutations, but to a greater degree [155,170,171]. Studies were able to differentiate between an HCM mutation and an RCM mutation at the same codon as the RCM mutation had greater calcium sensitivity of force and ATPase activity [155,171]. RCM mutations result in severely slow relaxation and short sarcomere lengths in cardiac myocytes which can lead to calcium buffering to the point of having increased baseline calcium levels in the cells [160,172,173,174]. In membrane-intact cardiac myocytes studies showed a continuum of HCM to RCM mutations, both showing slow relaxation with slow calcium decay kinetics with RCM mutations being slower [160]. At the whole animal and organ level, this can result in increased end-diastolic pressure and slow relaxation [172,174,175]. For some mutations in TnI, it has been shown that altered affinity to actin reduces the inhibition of myosin binding resulting in increased ATPase activity at low calcium levels [170]. These studies point to the extreme calcium sensitivity and inability to inhibit myosin as the mechanisms that lead to a stiff heart with significant diastolic dysfunction.

#### 5.1.3. Dilated Cardiomyopathy

DCM is a heterogeneous disease resulting in dilated ventricles with thin walls and systolic dysfunction [151,176,177]. DCM is a leading cause of heart failure and transplantation. Many factors can lead to DCM but for the purposes of this review, we will be discussing DCM caused by sarcomeric gene mutations. Monogenic sarcomeric-based DCM makes up about 25% of all causes of DCM [178]. Of the sarcomere proteins titin (TTN) is the most commonly mutated component although myosin, actin, Tm, TnT, and TnI mutations result in DCM as well [151,179,180]. Mutations resulting in DCM are at different locations than mutations which cause HCM or RCM [151,181]. Titin mutations are predominately truncations or deletions. Truncation mutations are thought to result in haploinsufficiency and loss of function, as a homozygous mutant gene expressed in human cells results in no sarcomere development and no titin [115,182]. Deletions and other mutations are thought to alter signaling or alter the passive stiffness of the sarcomere resulting in reduced sarcomere function. Myosin mutations tend to be at or near the nucleotide-binding site [183]. Most myosin mutations alter a step in the ATPase cycle resulting in accelerated detachment from actin leading to decreased contractility [151,154,183]. Thin filament mutations reduce calcium sensitivity of force development and result in reduced contractility and faster relaxation [164,184,185,186]. Animal models show dilated ventricles and systolic dysfunction with faster relaxation [187,188]. All these primary deficits in sarcomere function can lead to a weakened heart muscle and pathologic remodeling which presents as DCM in patients.

### 5.2. The Sarcomere as a Therapeutic Target

HCM, RCM, and DCM are the result of mutations in sarcomeric proteins giving rise to sarcomere dysfunction as the primary insult of the disease. As such, sarcomere-directed therapies could, in principle, alleviate the primary insult and allow for effective treatment. Studies have shown that targeting the sarcomere can be an effective treatment of these diseases [175,186]. For the purpose of this review, we will be discussing the clinically relevant small molecules targeting myosin. 

#### 5.2.1. Mavacamten and Aficamten

Mavacamten (originally MYK-461) was found in a high throughput screen of bovine myofibrils to reduce the maximal ATPase rate [189]. In cellular studies, it was found to reduce contractility in a calcium transient-independent manner making it a candidate therapeutic for HCM [189]. Further studies showed Mavacamten binds to myosin alone and alters the rate of Pi release in S1 preps [167]. Although this would result in total cycling rate reductions resulting in reduced ATPase activity, further experiments questioned if this was its only mechanism of action. Two studies revealed that Mavacamten stabilizes the SRX state of myosin. One showed biochemically that 2-headed heavy meromyosin (HMM) has an autoinhibited state compared to S1 which is stabilized by Mavacamten [190]. The second showed that reducing the S2 length in HMM reduced the number of heads in the SRX state [166]. Additionally, they showed that Mavacamten stabilizes the SRX only in the presence of the full S2 in HMM. This study also revealed the myosin mutations that lead to HCM destabilizing the SRX state and that Mavacamten can normalize this [166]. Further studies revealed that Mavacamten reduces hypertrophy if given before it presents in mutant myosin mouse models of HCM [189]. HCM mouse models for thin filament proteins demonstrated that Mavacamten can reduce calcium sensitivity and increase relaxation rates [191]. In addition, Mavacamten has been shown to reduce hypercontractility and slow relaxation in mouse and human models with MyBP-C mutations [167,192]. Mavacamten has been studied in several clinical trials and was recently approved for use in patients with HCM with left outflow tract obstructions [193]. 

Aficamten was chemically designed for therapeutic purposes from a myosin inhibitor found in a high throughput screen to reduce myofiber ATPase activity [194]. In cardiac myocytes Aficamten reduces contractility in a calcium independent manor and reduces fractional shortening in vivo similar to Mavacamten. Aficamten (IC 10: 0.8 μM to IC 50 7.9 μM) shows a larger effective concentration range than Mavacamten (IC 10: 0.6 μM to IC 50: 1.7 μM) in vivo, suggesting that it may have a safer range of dosing [194]. Aficamten has been in multiple clinical trials with promising preliminary results but is still in process of getting authorization [195]. 

#### 5.2.2. Omecamtiv Mercarbil and Danicamtiv

Omecamtiv mercarbil (OM) was derived from a molecule discovered in a high throughput screen as an activator of ATPase activity in cardiac myofibers. OM was shown to increase the ATPase activity of myosin alone, validating that myosin is the target in myofibers [196]. Early studies showed that myosin activated actin sliding velocity was slowed by OM, which clouded the interpretation of the early findings of increased ATPase activity. OM increased calcium sensitivity of cardiac myocytes and slowed skeletal muscle, but only at low calcium levels [197]. Indeed, two studies showed that OM inhibits the myosin working stroke and increases myosin binding to actin, resulting in an increased time of thin filament activation by myosin leading to increased force output [198,199]. In cardiac myocytes, OM increases contractility in a calcium transient-independent manner, although this is pacing-rate dependent [197,200]. OM increases fractional shortening in both rat and dog hearts in vivo [196]. OM was shown to increase calcium sensitivity in a genetic model of DCM with a Tm mutation suggesting it could be a potential treatment for sarcomeric DCM [201]. Multiple clinical trials for systolic heart failure have shown that OM has beneficial effects on heart performance, although small, and might be a potential therapy for patients with limited options for treatment [200,202].

Danicamtiv is a myosin activator with little published data on its mechanism of action. It is speculated to be cardiac myosin specific, increasing myosin’s strong binding to actin thus increasing force. Pre-clinical studies showed Danicamtiv to increase the ATPase activity of myofibrils and calcium sensitivity of tension in myofibers [203]. Danicamtiv improved systolic function in a dog model of heart failure [203]. In human-engineered tissue Danicamtiv showed increased systolic force in a dose-dependent manner, with less impact on relaxation than OM [204]. A clinical trial of Danicamtiv in systolic heart failure patients showed improved systolic function while having little impact on diastolic function [203]. 

## 6. Summary and Conclusions

Significant advances have been made to better understand the many factors that play a role in sarcomeric signaling. This review has centered the cardiac sarcomere as the main hub of cardiac myocyte intrinsic signaling, with the components that make up the sarcomere all playing instrumental roles. It is clear that these mechanisms of sarcomere activation and contraction incorporate levels of complexities, involving both the thin and thick filament, as well as proteins that bind to and interact with both. Collectively, this is reflected in the emerging focus of the field on inter-myofilament signaling. The ability to interrogate the roles of the sarcomeric proteins discussed here in an intact physiologically relevant system in real-time is a meaningful progression, building upon previous knowledge of sarcomere function derived from simplified systems under steady-state conditions. Examination of the influence of activating ligands, including calcium, TnC-TnI interactions, Tm positioning, myosin cycling, SRX-DRX states, load, and the influence of MyBP-C and titin, on cardiac muscle contraction using these advances will be able to deepen our understanding of sarcomere signaling processes. When directed to cardiac disease states, this increased knowledge will ultimately lead to the improved design of novel and impactful therapeutics. 

## Data Availability

Not Applicable.
